# Supplementary material for: Prediction of HLA genotypes from single-cell transcriptome data
Source: Front Immunol. 2023 Apr 25;14:1146826. doi: 10.3389/fimmu.2023.1146826 (PMC10167300; doi:10.3389/fimmu.2023.1146826)
Supplement: Supplementary file 1 [file DataSheet_1.pdf]

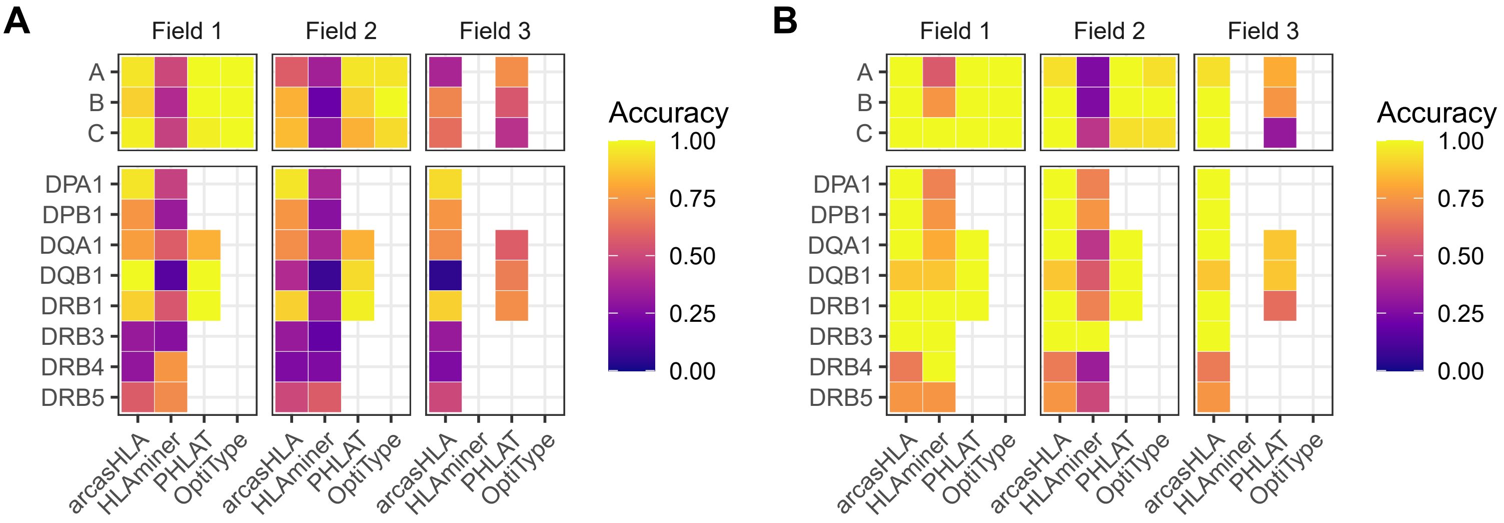

Supplemental Figure 1: Accuracy of HLA genotype predictions from alternate sequencing platforms

Mean accuracy of predicted genotypes compared to molecular genotyping from **A)** 3'-based scRNA-seq and **B)** paired-end bulk RNA-seq data

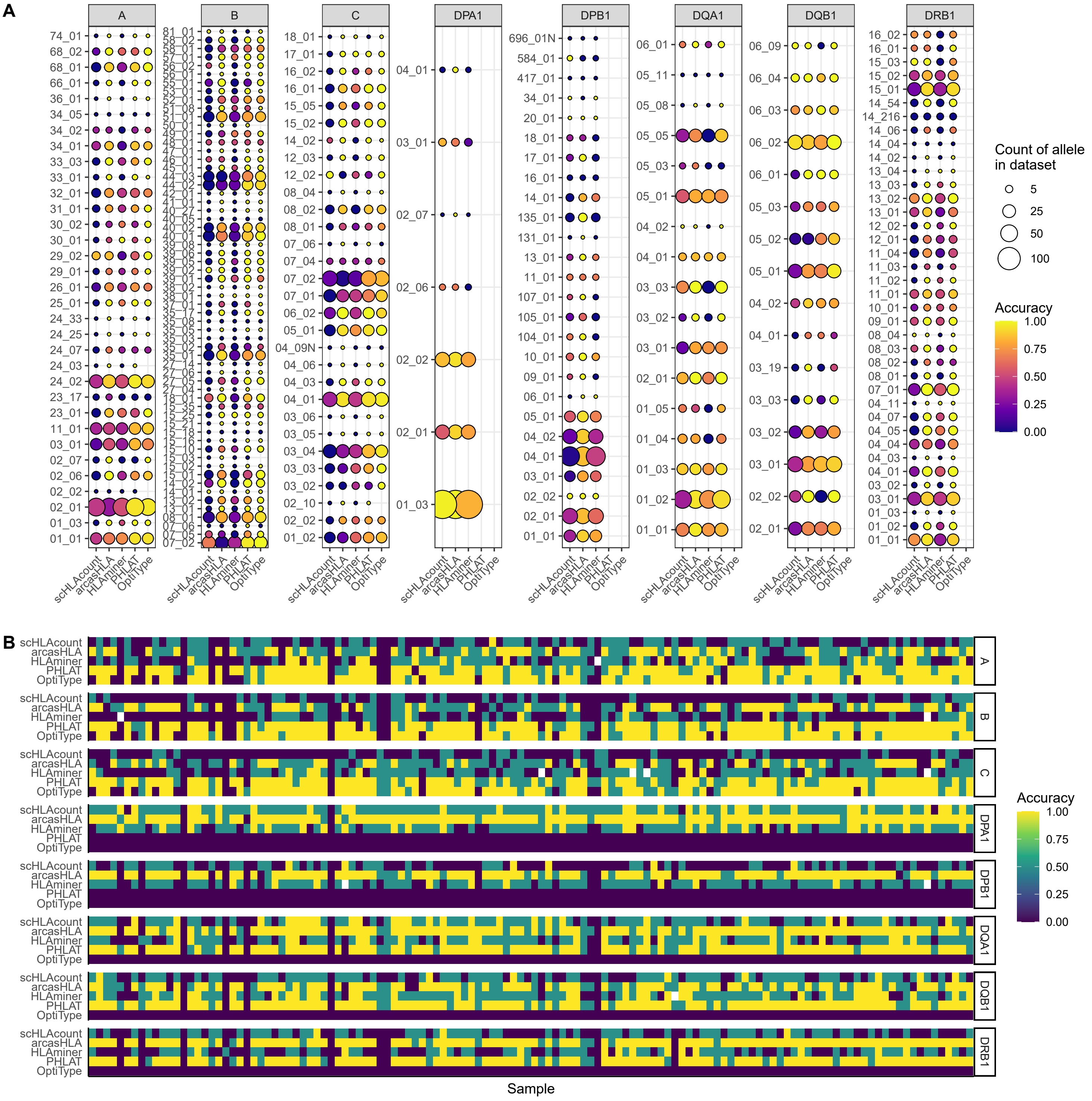

Supplemental Figure 2: Accuracy of HLA genotype predictions by allele and sample

A-B) 2-field accuracy of HLA genotype predictions. **A)** Mean accuracy based on identity of true allele from molecular genotyping. Size representative of count of allele throughout entire dataset. **B)** Accuracy of genotype prediction for each.

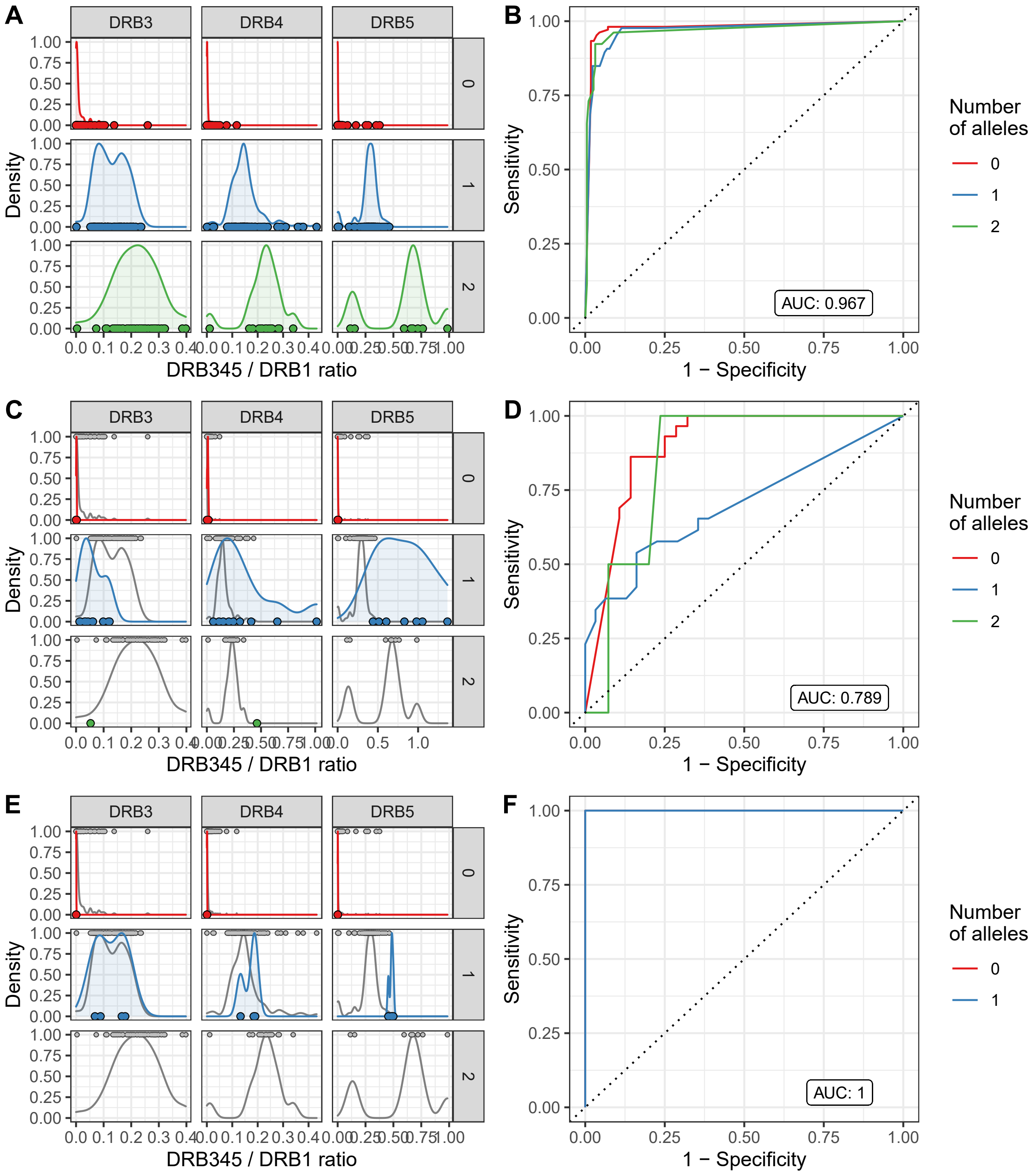

Supplemental Figure 3: HLA-DRB345 kNN classifier performance

A,C,E) Input DRB345 / DRB1 read ratios and **B,D,F)** kNN model performance ROCs from **A-B)** 5'-based scRNA-seq, **C-D)** 3'-based scRNA-seq, and **E-F)** paired-end bulk RNA-seq. Distribution of HLA-DRB345 : HLA-DRB1 read ratios by locus and ground truth allele copy number used by kNN classifier. Colored distributions represent sequencing platform as indicated above. Where present, grey represents 5'-based scRNA distribution as reference. Points represent ratios from individual samples overlaid along X-axis.

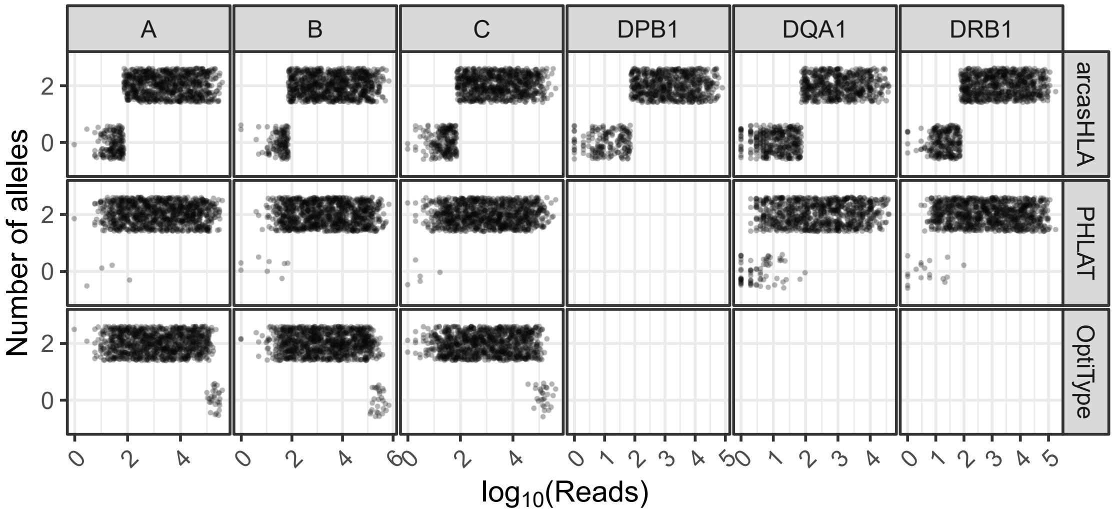

Supplemental Figure 4: Effect of sequence sampling depth on HLA predictions
Number of alleles identified based on number of reads aligned to the indicated locus. Raw sequencing data sampled to 100%, 10%, 1%, and 0.1% of original sample read total.

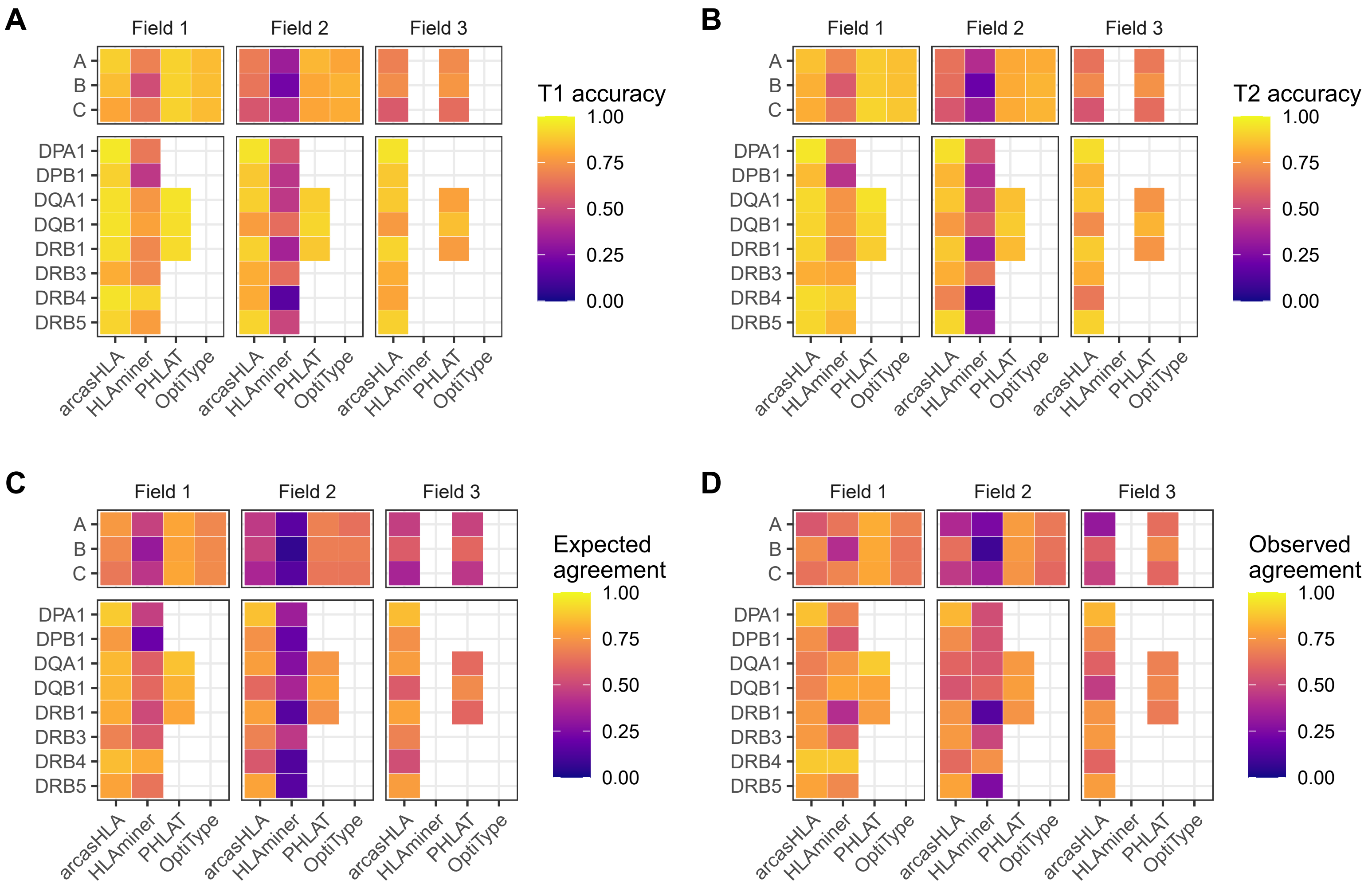

Supplemental Figure 5: Agreement between genotype predictions at different time points Mean accuracy of predicted genotypes compared to molecular genotyping at **A)** time point 1 and **B)** time point 2. **C)** Expected mean agreement between genotype predictions at type point 1 and type point 2 as represented by the joint probability of an accurate prediction at each time point. **D)** The observed mean agreement between predicted genotypes at time point 1 and time point 2.

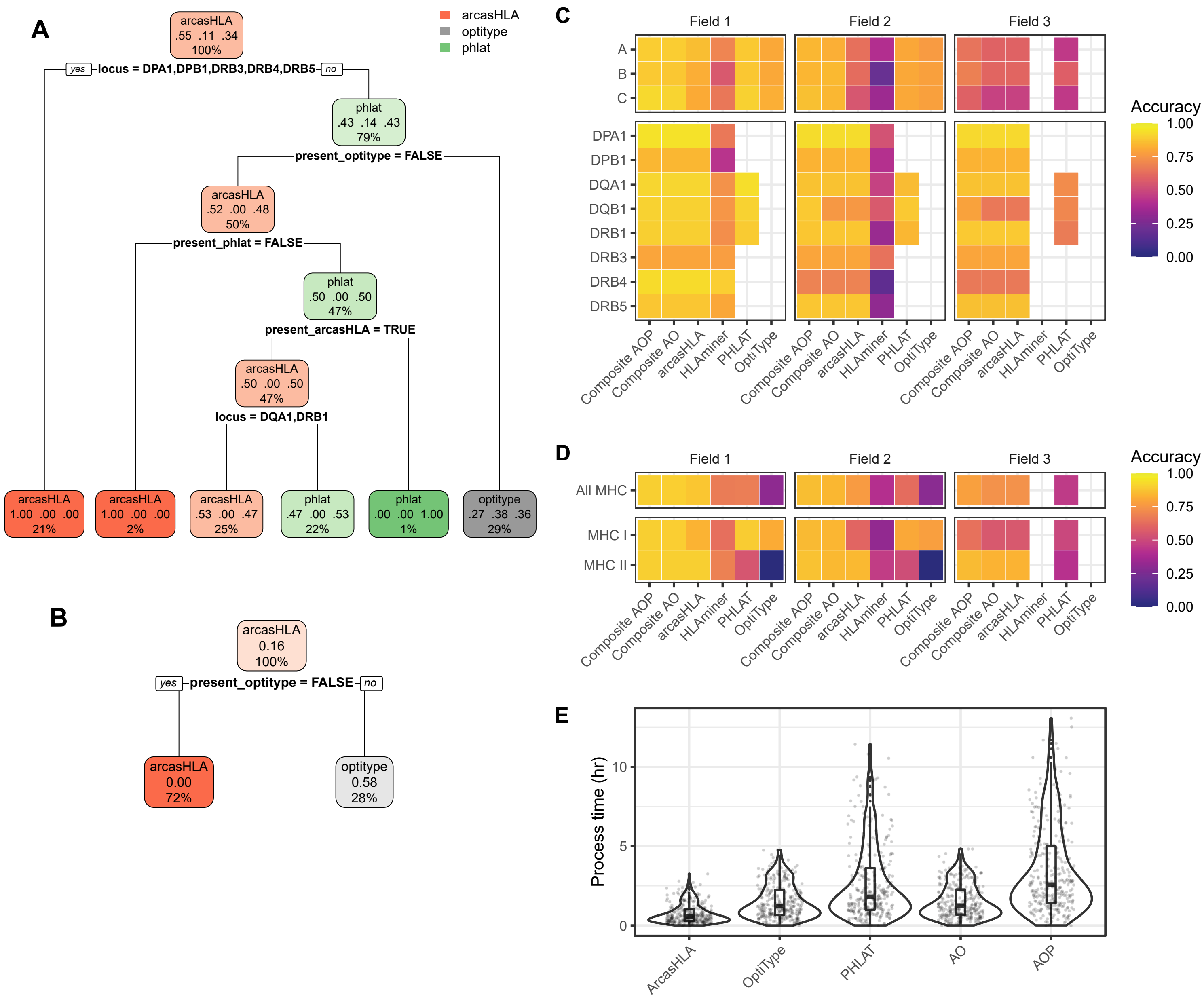

Supplemental Figure 6: Composite accuracy

A-B) Decision trees for identifying the highest accuracy genotyper based the set of available genotyper predictions, locus, and field. Trained on predictions from **A)** arcasHLA, OptiType, and PHLAT or **B)** only arcasHLA and OptiType. Tuned on 70% test set with 10-fold cross validation. AUC based on performance on 30% hold-out test set. **B-C)** Mean accuracy of individual and composite genotype predictions across **B)** individual loci and **D)** loci classes. **E)** Total runtime of individual and composite genotyping pipelines for each sample. Total runtime represents steps from mapping of all reads through genotyping of HLA loci.

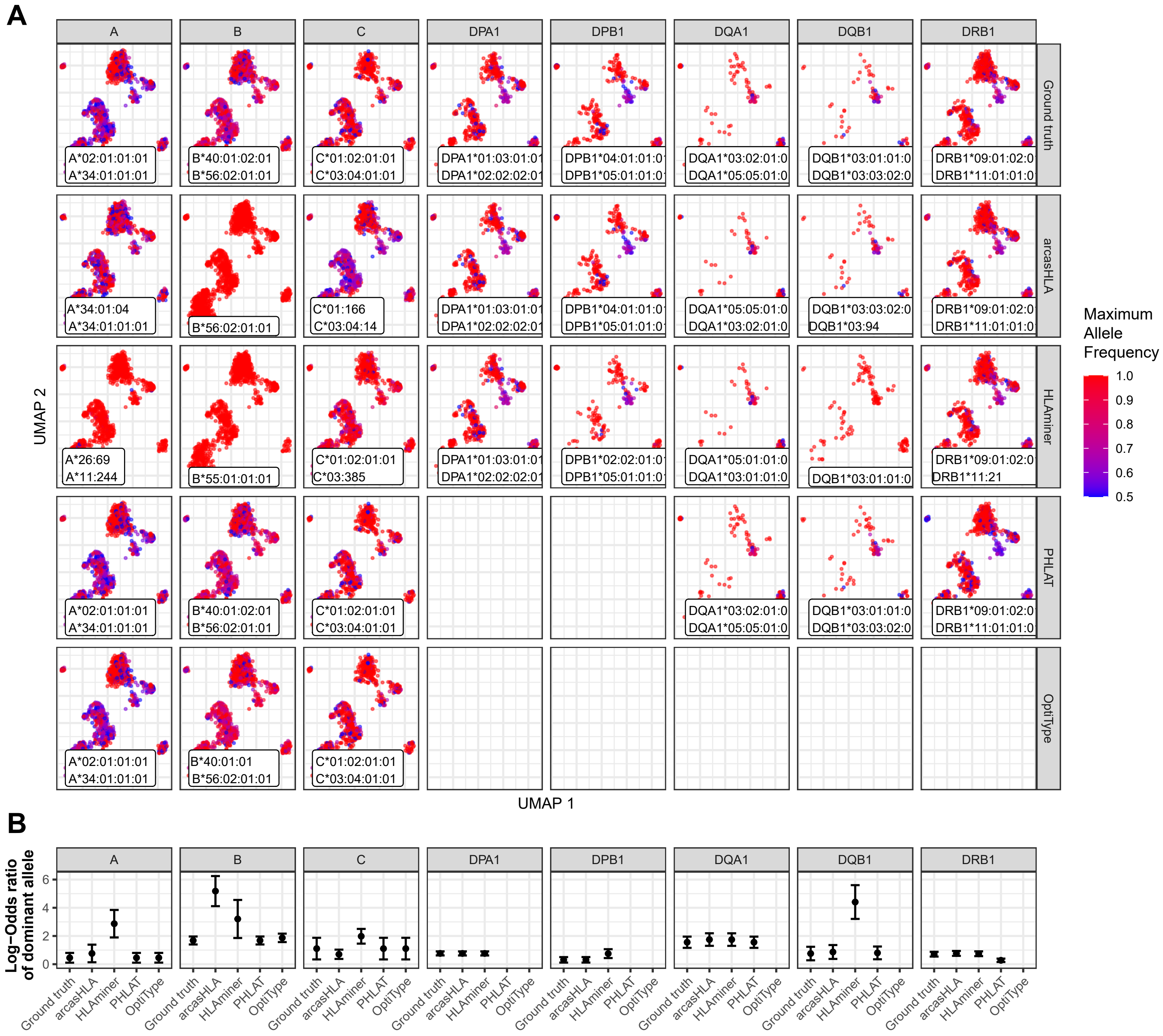

Supplemental Figure 7: Effect of genotype prediction accuracy on allele-specific expression ratios across HLA loci

(A-B) Same representative sample as included in *Figure 7*. **A)** UMAP plots colored by cell-specific expression frequency of the most highly expressed HLA allele determined by scHLAcount. The reference genotype from each genotyper used by scHLAcount is annotated. Columns specific HLA loci. Rows represent the specific genotyping algorithm used to determine the reference genotype supplied to scHLAcount to calculate allele specific expression. Blank plots represent HLA loci for which a specific genotyping algorithms does not generate predictions. **B)** For each HLA loci (rows)m summary log-odds ratios of dominant allele across all cells in cDC cluster determined by random effects model. Error bars represent summary standard error.

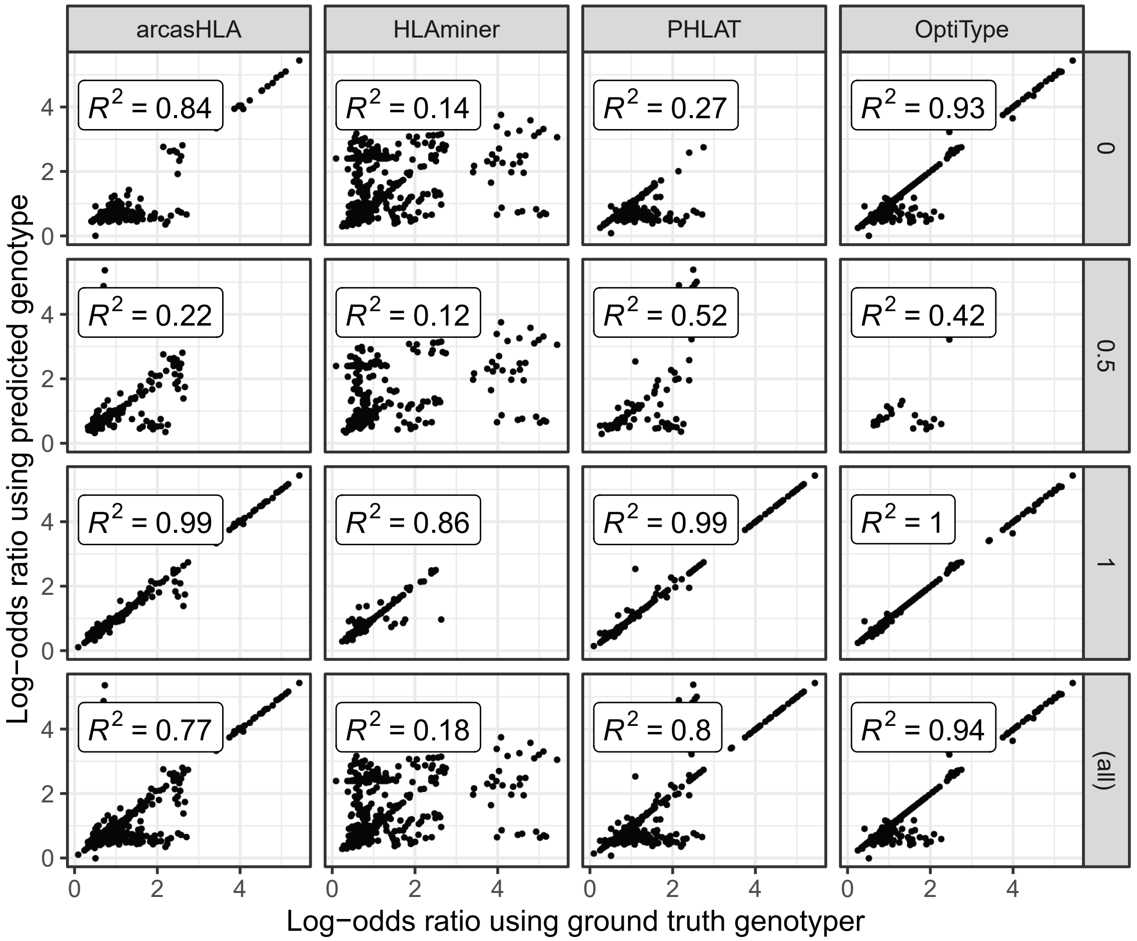

Supplemental Figure 8: Effect of genotype prediction accuracy on accuracy of HLA-A allele specific expression ratios

Correlation of ground truth- and genotyper-derived HLA-A allele log-odds ratio from all samples and cell types. Rows reflect correlations stratified by underlying genotype prediction accuracy, as well as final row containing all predictions.
