# Supplementary material for: Prediction of HLA genotypes from single-cell transcriptome data
Source: Front Immunol. 2023 Apr 25;14:1146826. doi: 10.3389/fimmu.2023.1146826 (PMC10167300; doi:10.3389/fimmu.2023.1146826)
Supplement: Supplementary file 2 [file DataSheet_2.pdf]

	Field 1					Field 2					Field 3		
	scHLAcount	arcasHLA	HLAminer	PHLAT	OptiType	scHLAcount	arcasHLA	HLAminer	PHLAT	OptiType	scHLAcount	arcasHLA	PHLAT
A	0.68 ± 0.04	0.86 ± 0.03	0.71 ± 0.03	0.89 ± 0.03	0.92 ± 0.02	0.44 ± 0.04	0.64 ± 0.03	0.41 ± 0.03	0.81 ± 0.03	0.87 ± 0.03	0.06 ± 0.02	0.79 ± 0.03	0.8 ± 0.03
B	0.44 ± 0.04	0.82 ± 0.02	0.56 ± 0.03	0.88 ± 0.02	0.92 ± 0.02	0.21 ± 0.03	0.63 ± 0.03	0.2 ± 0.03	0.81 ± 0.03	0.9 ± 0.02	0.2 ± 0.03	0.8 ± 0.03	0.83 ± 0.03
C	0.82 ± 0.03	0.82 ± 0.02	0.66 ± 0.02	0.91 ± 0.02	0.94 ± 0.01	0.16 ± 0.03	0.55 ± 0.03	0.35 ± 0.03	0.81 ± 0.03	0.9 ± 0.02	0.02 ± 0.01	0.64 ± 0.03	0.82 ± 0.03
DPA1	0.71 ± 0.02	0.95 ± 0.02	0.66 ± 0.02	—	—	0.69 ± 0.02	0.94 ± 0.02	0.54 ± 0.03	—	—	0.65 ± 0.03	0.94 ± 0.02	—
DPB1	0.25 ± 0.03	0.85 ± 0.03	0.43 ± 0.02	—	—	0.25 ± 0.03	0.84 ± 0.03	0.42 ± 0.02	—	—	0.34 ± 0.04	0.84 ± 0.03	—
DQA1	0.81 ± 0.03	0.92 ± 0.02	0.85 ± 0.02	0.94 ± 0.01	—	0.55 ± 0.03	0.88 ± 0.02	0.55 ± 0.03	0.86 ± 0.03	—	0.71 ± 0.04	0.88 ± 0.02	0.86 ± 0.03
DQB1	0.83 ± 0.03	0.91 ± 0.02	0.88 ± 0.02	0.91 ± 0.02	—	0.54 ± 0.04	0.76 ± 0.03	0.67 ± 0.03	0.89 ± 0.02	—	0.59 ± 0.04	0.78 ± 0.03	0.87 ± 0.02
DRB1	0.7 ± 0.04	0.9 ± 0.02	0.73 ± 0.03	0.89 ± 0.03	—	0.48 ± 0.04	0.88 ± 0.03	0.34 ± 0.03	0.85 ± 0.03	—	0.49 ± 0.04	0.88 ± 0.03	0.78 ± 0.03

Supplemental Table 1 - 5'-based scRNA-seq success
Success of predicted genotypes from 5'-based scRNA-seq sequences. Values represent mean success +/- SEM

	Field 1					Field 2					Field 3		
	scHLAcount	arcasHLA	HLAminer	PHLAT	OptiType	scHLAcount	arcasHLA	HLAminer	PHLAT	OptiType	scHLAcount	arcasHLA	PHLAT
A	0.41 ± 0.03	0.86 ± 0.03	0.7 ± 0.03	0.88 ± 0.03	0.81 ± 0.03	0.24 ± 0.02	0.63 ± 0.03	0.4 ± 0.03	0.8 ± 0.03	0.76 ± 0.04	0.02 ± 0.01	0.59 ± 0.03	0.44 ± 0.04
B	0.26 ± 0.02	0.82 ± 0.03	0.56 ± 0.03	0.88 ± 0.02	0.81 ± 0.03	0.13 ± 0.02	0.62 ± 0.03	0.2 ± 0.03	0.81 ± 0.03	0.78 ± 0.03	0.07 ± 0.02	0.6 ± 0.03	0.58 ± 0.04
C	0.48 ± 0.02	0.81 ± 0.02	0.66 ± 0.02	0.9 ± 0.02	0.82 ± 0.03	0.1 ± 0.02	0.55 ± 0.03	0.34 ± 0.03	0.81 ± 0.03	0.78 ± 0.03	0.01 ± 0.01	0.47 ± 0.03	0.44 ± 0.04
DPA1	0.62 ± 0.02	0.94 ± 0.02	0.66 ± 0.02	—	—	0.6 ± 0.02	0.93 ± 0.02	0.53 ± 0.03	—	—	0.56 ± 0.03	0.92 ± 0.02	—
DPB1	0.21 ± 0.03	0.84 ± 0.03	0.42 ± 0.02	—	—	0.21 ± 0.03	0.83 ± 0.03	0.41 ± 0.02	—	—	0.21 ± 0.03	0.83 ± 0.03	—
DQA1	0.72 ± 0.03	0.91 ± 0.02	0.74 ± 0.03	0.93 ± 0.02	—	0.48 ± 0.03	0.87 ± 0.02	0.47 ± 0.03	0.85 ± 0.03	—	0.45 ± 0.03	0.87 ± 0.02	0.72 ± 0.03
DQB1	0.53 ± 0.02	0.9 ± 0.02	0.75 ± 0.03	0.9 ± 0.02	—	0.35 ± 0.03	0.75 ± 0.03	0.56 ± 0.03	0.88 ± 0.02	—	0.3 ± 0.03	0.66 ± 0.03	0.7 ± 0.03
DRB1	0.4 ± 0.02	0.9 ± 0.03	0.72 ± 0.03	0.88 ± 0.03	—	0.28 ± 0.02	0.87 ± 0.03	0.33 ± 0.03	0.84 ± 0.03	—	0.29 ± 0.03	0.88 ± 0.03	0.67 ± 0.04

Supplemental Table 2 - 5'-based scRNA-seq accuracy
Accuracy of predicted genotypes from 5'-based scRNA-seq sequences. Values represent mean accuracy +/- SEM

	Field 1				Field 2				Field 3	
	arcasHLA	HLAminer	PHLAT	OptiType	arcasHLA	HLAminer	PHLAT	OptiType	arcasHLA	PHLAT
A	0.95 ± 0.03	0.5 ± 0.06	1 ± 0	1 ± 0	0.58 ± 0.07	0.35 ± 0.07	0.95 ± 0.03	0.95 ± 0.03	0.38 ± 0.06	0.72 ± 0.08
B	0.9 ± 0.04	0.4 ± 0.04	1 ± 0	1 ± 0	0.82 ± 0.05	0.2 ± 0.05	0.9 ± 0.05	1 ± 0	0.7 ± 0.06	0.55 ± 0.09
C	0.98 ± 0.02	0.48 ± 0.08	0.98 ± 0.02	1 ± 0	0.85 ± 0.06	0.3 ± 0.06	0.82 ± 0.05	0.92 ± 0.04	0.62 ± 0.06	0.42 ± 0.07
DPA1	0.95 ± 0.03	0.48 ± 0.02	—	—	0.95 ± 0.03	0.38 ± 0.04	—	—	0.92 ± 0.05	—
DPB1	0.75 ± 0.05	0.32 ± 0.06	—	—	0.75 ± 0.05	0.28 ± 0.05	—	—	0.75 ± 0.05	—
DQA1	0.78 ± 0.05	0.58 ± 0.04	0.82 ± 0.05	—	0.72 ± 0.06	0.38 ± 0.06	0.82 ± 0.05	—	0.72 ± 0.06	0.58 ± 0.09
DQB1	1 ± 0	0.15 ± 0.06	1 ± 0	—	0.4 ± 0.06	0.07 ± 0.04	0.92 ± 0.04	—	0.05 ± 0.03	0.68 ± 0.07
DRB1	0.9 ± 0.04	0.55 ± 0.06	1 ± 0	—	0.9 ± 0.04	0.32 ± 0.07	0.98 ± 0.02	—	0.9 ± 0.04	0.72 ± 0.08
DRB3	0.32 ± 0.11	0.27 ± 0.11	—	—	0.32 ± 0.11	0.18 ± 0.09	—	—	0.32 ± 0.11	—
DRB4	0.3 ± 0.1	0.75 ± 0.1	—	—	0.25 ± 0.08	0.25 ± 0.1	—	—	0.25 ± 0.08	—
DRB5	0.57 ± 0.08	0.71 ± 0.09	—	—	0.5 ± 0.09	0.57 ± 0.1	—	—	0.5 ± 0.09	—

Supplemental Table 3 - 3'-based scRNA-seq accuracy

Accuracy of predicted genotypes from 3'-based scRNA-seq sequences. Values represent mean accuracy +/- SEM

	Field 1				Field 2				Field 3	
	arcasHLA	HLAminer	PHLAT	OptiType	arcasHLA	HLAminer	PHLAT	OptiType	arcasHLA	PHLAT
A	1 ± 0	0.56 ± 0.06	1 ± 0	1 ± 0	0.94 ± 0.06	0.25 ± 0.09	1 ± 0	0.94 ± 0.06	0.94 ± 0.06	0.81 ± 0.13
B	1 ± 0	0.75 ± 0.09	1 ± 0	1 ± 0	1 ± 0	0.25 ± 0.13	1 ± 0	1 ± 0	1 ± 0	0.75 ± 0.16
C	1 ± 0	1 ± 0	1 ± 0	1 ± 0	1 ± 0	0.44 ± 0.11	0.94 ± 0.06	0.94 ± 0.06	1 ± 0	0.31 ± 0.16
DPA1	1 ± 0	0.69 ± 0.09	—	—	1 ± 0	0.69 ± 0.09	—	—	1 ± 0	—
DPB1	1 ± 0	0.75 ± 0.09	—	—	1 ± 0	0.75 ± 0.09	—	—	1 ± 0	—
DQA1	1 ± 0	0.81 ± 0.09	1 ± 0	—	1 ± 0	0.44 ± 0.15	1 ± 0	—	1 ± 0	0.88 ± 0.08
DQB1	0.88 ± 0.08	0.88 ± 0.08	1 ± 0	—	0.88 ± 0.08	0.56 ± 0.11	1 ± 0	—	0.88 ± 0.08	0.88 ± 0.08
DRB1	1 ± 0	1 ± 0	1 ± 0	—	1 ± 0	0.69 ± 0.09	1 ± 0	—	1 ± 0	0.62 ± 0.16
DRB3	1 ± 0	1 ± 0	—	—	1 ± 0	1 ± 0	—	—	1 ± 0	—
DRB4	0.67 ± 0.1	1 ± 0	—	—	0.67 ± 0.1	0.33 ± 0.2	—	—	0.67 ± 0.1	—
DRB5	0.75 ± 0.18	0.75 ± 0.18	—	—	0.75 ± 0.18	0.5 ± 0.2	—	—	0.75 ± 0.18	—

Supplemental Table 4 - Paired-end bulkRNA-seq accuracy
Accuracy of predicted genotypes from bulk RNA-seq sequences. Values represent mean accuracy +/- SEM

	Field 1				Field 2				Field 3			
	arcasHLA		HLAminer		arcasHLA		HLAminer		arcasHLA		HLAminer	
	Unfiltered	Filtered	Unfiltered	Filtered	Unfiltered	Filtered	Unfiltered	Filtered	Unfiltered	Filtered	Unfiltered	Filtered
DRB3	0.46 ± 0.03	0.79 ± 0.04	0.44 ± 0.03	0.78 ± 0.03	0.46 ± 0.03	0.79 ± 0.04	0.35 ± 0.03	0.65 ± 0.04	0.46 ± 0.03	0.79 ± 0.04	—	—
DRB4	0.44 ± 0.02	0.91 ± 0.02	0.77 ± 0.04	0.88 ± 0.03	0.4 ± 0.02	0.69 ± 0.04	0.14 ± 0.03	0.17 ± 0.03	0.39 ± 0.03	0.66 ± 0.04	—	—
DRB5	0.35 ± 0.03	0.9 ± 0.03	0.33 ± 0.03	0.83 ± 0.03	0.35 ± 0.03	0.88 ± 0.03	0.15 ± 0.03	0.33 ± 0.04	0.33 ± 0.03	0.86 ± 0.03	—	—

Supplemental Table 5 - *HLA-DRB345* accuracy

Accuracy of *HLA-DRB345* genotypes filtered or unfiltered by KNN copy number classifier. Values represent mean accuracy +/- SEM

	Field 1								Field 2								Field 3			
	arcasHLA		HLAminer		PHLAT		OptiType		arcasHLA		HLAminer		PHLAT		OptiType		arcasHLA		PHLAT	
	T1	T2	T1	T2	T1	T2	T1	T2	T1	T2	T1	T2	T1	T2	T1	T2	T1	T2	T1	T2
A	0.9 ± 0.02	0.86 ± 0.03	0.68 ± 0.03	0.7 ± 0.03	0.9 ± 0.02	0.89 ± 0.03	0.85 ± 0.03	0.86 ± 0.03	0.67 ± 0.03	0.64 ± 0.03	0.33 ± 0.03	0.41 ± 0.03	0.84 ± 0.03	0.81 ± 0.03	0.8 ± 0.03	0.81 ± 0.03	0.68 ± 0.04	0.64 ± 0.04	0.72 ± 0.04	0.66 ± 0.04
B	0.86 ± 0.02	0.82 ± 0.02	0.52 ± 0.03	0.56 ± 0.03	0.9 ± 0.02	0.88 ± 0.02	0.86 ± 0.03	0.86 ± 0.03	0.65 ± 0.03	0.63 ± 0.03	0.22 ± 0.03	0.2 ± 0.03	0.8 ± 0.03	0.81 ± 0.03	0.83 ± 0.03	0.83 ± 0.03	0.72 ± 0.04	0.71 ± 0.04	0.75 ± 0.04	0.74 ± 0.04
C	0.8 ± 0.03	0.82 ± 0.02	0.67 ± 0.03	0.66 ± 0.02	0.9 ± 0.02	0.91 ± 0.02	0.85 ± 0.03	0.88 ± 0.03	0.55 ± 0.03	0.55 ± 0.03	0.41 ± 0.03	0.35 ± 0.03	0.8 ± 0.03	0.81 ± 0.03	0.81 ± 0.03	0.83 ± 0.03	0.56 ± 0.04	0.54 ± 0.03	0.62 ± 0.04	0.62 ± 0.04
DPA1	0.96 ± 0.01	0.95 ± 0.02	0.66 ± 0.02	0.66 ± 0.02	—	—	—	—	0.95 ± 0.01	0.94 ± 0.02	0.55 ± 0.03	0.54 ± 0.03	—	—	—	—	0.94 ± 0.01	0.93 ± 0.02	—	—
DPB1	0.9 ± 0.02	0.85 ± 0.03	0.44 ± 0.03	0.43 ± 0.02	—	—	—	—	0.88 ± 0.02	0.84 ± 0.03	0.44 ± 0.03	0.41 ± 0.02	—	—	—	—	0.88 ± 0.02	0.83 ± 0.03	—	—
DQA1	0.94 ± 0.02	0.92 ± 0.02	0.75 ± 0.02	0.74 ± 0.02	0.95 ± 0.02	0.94 ± 0.01	—	—	0.9 ± 0.02	0.88 ± 0.02	0.45 ± 0.03	0.47 ± 0.03	0.89 ± 0.02	0.86 ± 0.03	—	—	0.88 ± 0.03	0.88 ± 0.02	0.78 ± 0.03	0.74 ± 0.03
DQB1	0.94 ± 0.02	0.91 ± 0.02	0.79 ± 0.02	0.75 ± 0.03	0.93 ± 0.02	0.91 ± 0.02	—	—	0.77 ± 0.03	0.76 ± 0.03	0.62 ± 0.03	0.56 ± 0.03	0.91 ± 0.02	0.89 ± 0.02	—	—	0.76 ± 0.03	0.72 ± 0.03	0.86 ± 0.03	0.83 ± 0.03
DRB1	0.93 ± 0.02	0.9 ± 0.02	0.71 ± 0.03	0.73 ± 0.03	0.93 ± 0.02	0.89 ± 0.03	—	—	0.9 ± 0.02	0.88 ± 0.03	0.36 ± 0.03	0.34 ± 0.03	0.88 ± 0.03	0.85 ± 0.03	—	—	0.91 ± 0.02	0.89 ± 0.03	0.77 ± 0.03	0.75 ± 0.03
DRB3	0.82 ± 0.04	0.82 ± 0.04	0.71 ± 0.05	0.79 ± 0.04	—	—	—	—	0.82 ± 0.04	0.82 ± 0.04	0.62 ± 0.05	0.66 ± 0.05	—	—	—	—	0.82 ± 0.04	0.82 ± 0.04	—	—
DRB4	0.95 ± 0.03	0.92 ± 0.03	0.91 ± 0.03	0.89 ± 0.03	—	—	—	—	0.81 ± 0.05	0.69 ± 0.06	0.15 ± 0.05	0.17 ± 0.05	—	—	—	—	0.79 ± 0.05	0.66 ± 0.06	—	—
DRB5	0.91 ± 0.04	0.91 ± 0.04	0.77 ± 0.06	0.84 ± 0.05	—	—	—	—	0.91 ± 0.04	0.91 ± 0.04	0.49 ± 0.07	0.33 ± 0.07	—	—	—	—	0.9 ± 0.05	0.9 ± 0.04	—	—

Supplemental Table 6 - Reproducibility of genotype prediction accuracy

Accuracy of predicted genotypes from repeated sampling of individuals at time point 1 (T1) and time point 2 (T2). Values represent mean accuracy +/- SEM

	Field 1						Field 2						Field 3			
	Composite AOP	Composite AO	arcasHLA	HLAminer	PHLAT	OptiType	Composite AOP	Composite AO	arcasHLA	HLAminer	PHLAT	OptiType	Composite AOP	Composite AO	arcasHLA	PHLAT
MHC All	0.9 ± 0.02	0.89 ± 0.02	0.87 ± 0.02	0.67 ± 0.01	0.67 ± 0.01	0.3 ± 0.01	0.86 ± 0.02	0.84 ± 0.02	0.76 ± 0.02	0.41 ± 0.01	0.62 ± 0.02	0.29 ± 0.01	0.78 ± 0.02	0.74 ± 0.02	0.74 ± 0.02	0.45 ± 0.02
MHC I	0.9 ± 0.02	0.89 ± 0.02	0.83 ± 0.02	0.63 ± 0.02	0.89 ± 0.02	0.81 ± 0.03	0.85 ± 0.02	0.84 ± 0.02	0.6 ± 0.02	0.31 ± 0.02	0.8 ± 0.02	0.78 ± 0.03	0.64 ± 0.02	0.56 ± 0.02	0.56 ± 0.02	0.49 ± 0.03
MHC II	0.9 ± 0.02	0.9 ± 0.02	0.9 ± 0.02	0.68 ± 0.02	0.54 ± 0.01	0 ± 0	0.87 ± 0.02	0.84 ± 0.02	0.84 ± 0.02	0.46 ± 0.02	0.52 ± 0.01	0 ± 0	0.85 ± 0.02	0.83 ± 0.02	0.83 ± 0.02	0.42 ± 0.01
A	0.89 ± 0.03	0.89 ± 0.03	0.86 ± 0.03	0.7 ± 0.03	0.88 ± 0.03	0.81 ± 0.03	0.84 ± 0.03	0.82 ± 0.03	0.63 ± 0.03	0.4 ± 0.03	0.8 ± 0.03	0.76 ± 0.04	0.64 ± 0.03	0.59 ± 0.03	0.59 ± 0.03	0.44 ± 0.04
B	0.88 ± 0.02	0.88 ± 0.02	0.82 ± 0.03	0.56 ± 0.03	0.88 ± 0.02	0.81 ± 0.03	0.84 ± 0.03	0.85 ± 0.03	0.62 ± 0.03	0.2 ± 0.03	0.81 ± 0.03	0.78 ± 0.03	0.68 ± 0.03	0.6 ± 0.03	0.6 ± 0.03	0.58 ± 0.04
C	0.92 ± 0.02	0.91 ± 0.02	0.81 ± 0.02	0.66 ± 0.02	0.9 ± 0.02	0.82 ± 0.03	0.86 ± 0.02	0.84 ± 0.02	0.55 ± 0.03	0.34 ± 0.03	0.81 ± 0.03	0.78 ± 0.03	0.59 ± 0.03	0.47 ± 0.03	0.47 ± 0.03	0.44 ± 0.04
DPA1	0.94 ± 0.02	0.94 ± 0.02	0.94 ± 0.02	0.66 ± 0.02	—	—	0.93 ± 0.02	0.93 ± 0.02	0.93 ± 0.02	0.53 ± 0.03	—	—	0.92 ± 0.02	0.92 ± 0.02	0.92 ± 0.02	—
DPB1	0.84 ± 0.03	0.84 ± 0.03	0.84 ± 0.03	0.42 ± 0.02	—	—	0.83 ± 0.03	0.83 ± 0.03	0.83 ± 0.03	0.41 ± 0.02	—	—	0.83 ± 0.03	0.83 ± 0.03	0.83 ± 0.03	—
DQA1	0.91 ± 0.02	0.91 ± 0.02	0.91 ± 0.02	0.74 ± 0.03	0.93 ± 0.02	—	0.87 ± 0.02	0.87 ± 0.02	0.87 ± 0.02	0.47 ± 0.03	0.85 ± 0.03	—	0.87 ± 0.02	0.87 ± 0.02	0.87 ± 0.02	0.72 ± 0.03
DQB1	0.9 ± 0.02	0.9 ± 0.02	0.9 ± 0.02	0.75 ± 0.03	0.9 ± 0.02	—	0.88 ± 0.02	0.75 ± 0.03	0.75 ± 0.03	0.56 ± 0.03	0.88 ± 0.02	—	0.79 ± 0.03	0.66 ± 0.03	0.66 ± 0.03	0.7 ± 0.03
DRB1	0.9 ± 0.03	0.9 ± 0.03	0.9 ± 0.03	0.72 ± 0.03	0.88 ± 0.03	—	0.87 ± 0.03	0.87 ± 0.03	0.87 ± 0.03	0.33 ± 0.03	0.84 ± 0.03	—	0.88 ± 0.03	0.88 ± 0.03	0.88 ± 0.03	0.67 ± 0.04
DRB3	0.8 ± 0.04	0.8 ± 0.04	0.8 ± 0.04	0.77 ± 0.03	—	—	0.8 ± 0.04	0.8 ± 0.04	0.8 ± 0.04	0.64 ± 0.04	—	—	0.8 ± 0.04	0.8 ± 0.04	0.8 ± 0.04	—
DRB4	0.92 ± 0.02	0.92 ± 0.02	0.92 ± 0.02	0.89 ± 0.02	—	—	0.69 ± 0.04	0.69 ± 0.04	0.69 ± 0.04	0.17 ± 0.03	—	—	0.66 ± 0.04	0.66 ± 0.04	0.66 ± 0.04	—
DRB5	0.88 ± 0.03	0.88 ± 0.03	0.88 ± 0.03	0.8 ± 0.03	—	—	0.88 ± 0.03	0.88 ± 0.03	0.88 ± 0.03	0.31 ± 0.04	—	—	0.86 ± 0.03	0.86 ± 0.03	0.86 ± 0.03	—

Supplemental Table 7 - Composite genotype accuracy
Success of predicted genotypes from using decision tree-based composites. AOP = arcasHLA, OptiType, and PHLAT, AO = arcasHLA and OptiType. Values represent mean accuracy +/- SEM
